# The role of employability capitals in supporting nutrition science graduate outcomes: an international qualitative study

**DOI:** 10.1017/S1368980024001411

**Published:** 2024-09-26

**Authors:** Sarah O’Donovan, Charlotte Barber, Claire Palermo, Lisa Ryan

**Affiliations:** 1 Department of Sport, Exercise and Nutrition, Atlantic Technological University, Galway H91 T8NW, Ireland; 2 Department of Nutrition, Dietetics and Food, Monash University, Melbourne, Australia

**Keywords:** Nutrition workforce, Graduate capital model, Graduate employability, Nutrition graduates

## Abstract

**Objective:**

The study sought to explore nutrition graduates’ employability and role of employability capitals in supporting nutrition science graduate outcomes.

**Design:**

In-depth semi-structured, audio-recorded interviews were conducted with nutrition graduates who had completed a nutrition science degree between 2015 and 2021. Interpretivism guided this study, which endeavoured to co-construct meaning with participants. Transcribed interviews were thematically analysed, whereby data were coded, themes identified and discussed by all authors. The data were further mapped against the graduate capital model (GCM) by deductively coding against the five graduate capitals (human, identity, social, psychological and cultural).

**Setting:**

Ireland and Australia.

**Participants:**

Forty-two nutrition graduates from across nine universities in Ireland and twenty-two from a single university programme in Australia.

**Results:**

All elements of the GCM were identified with human, social and identity capital most dominant and identified as significantly influential on employability. Presence or absence of these capitals could be clearly identified within each graduates’ experience. Formation of professional identity and connection to the profession was strongest amongst Irish graduates. However, more than half of the Australian cohort perceived barriers to professional identity formation, including lack of regulation, imposter syndrome, presence of non-qualified individuals and comparison to dietetics. Both psychological and cultural capitals were rarely spoken about.

**Conclusion:**

The development of human, social and identity capital is observed among nutrition science graduates. Further investigation is required to enhance the process of identity development and ascertain potential remedies for obstacles. The absence of psychological and cultural capital, therefore, poses a significant issue for the resilience and comprehension of prospective graduates.

The prevalence of diet-related chronic diseases across the world is high^(1)^. Together with a growing emphasis on sustainable food production and food security, this necessitates deliberate action. Although nutrition science graduates are well positioned to address these challenges, it remains uncertain whether their potential is being fully realised^(2)^. Nutrition science graduates typically complete 3 to 4 years of undergraduate study in nutrition-related sciences and are qualified to work as researchers and nutritionists. These nutrition-related science undergraduate degrees include human nutrition, nutritional science, public health nutrition, and nutrition and health science. Research on the employability and employment outcomes of nutrition science graduates at one Australian university reported that building employability skills, such as communication and business skills, in the curriculum through the development of professional social networks and professional identity may be important in improving the employability of nutrition science graduates^(3)^. However, little is known in a global context about nutrition science graduate employability, employment outcomes and workforce readiness.

Connecting the employability of nutrition science graduates with associated theoretical models of employability may provide a more comprehensive understanding of the factors that shape successful employment outcomes. The graduate capital model (GCM) is one such meso-theory, which suggests that individuals can enhance their career prospects by developing a variety of employability ‘capitals’, defined as important resources that provide an advantage in the labour market^(4)^. To date, the GCM has not been applied to understand nutrition science graduate employability. The model supports the notion that employability goes beyond grades^(5)^. Resources in this model include human, social, identity, psychological and cultural capital (Fig. 1). These resources span the dimensions of education, social, culture and psycho-social factors and are acquired both within and outside formal education. Employer perceptions of what constitutes a ‘stand out’ candidate appear to align with the GCM. Anderson and Tomlinson (2021) reported that employers value talent and creativity in candidates, and that generic business knowledge, interpersonal competencies and personal qualities are important^(5)^. Such personal qualities that are said to make a candidate stand out include resilience, adaptability, flexibility and confidence in their own abilities (i.e. psychological capital). Crowley and Jeske (2021) also explored recruiter perceptions and expectations of graduate attributes, determining that ‘fit’ and formal qualifications were of the utmost importance to employers (i.e. human and cultural capital)^(6)^. Employee ‘fit’ was determined by desirable attributes such as commitment, motivation and goal orientation, as well as openness to learning and flexibility. The model is proposed as an alternative to ‘skills’ or ‘attitudes’ graduate outcomes approaches, suggesting that it is not the acquisition of capabilities that enhance employment outcomes, but rather graduates’ life and education experiences together with the labour market that shapes employment outcomes.

**Fig. 1:**
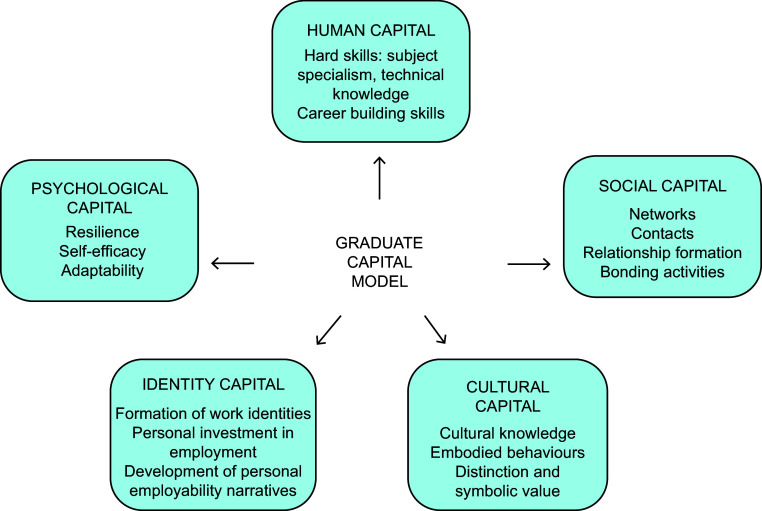
Description of the graduate capital model (Adapted from Tomlinson 2017, with permission)^(4)^

With an understanding of nutrition science graduate employability, educators can better support graduates to find and secure meaningful work and contribute to the world’s social agenda. Integrating the role of employability capital in this discussion also has important implications for nutrition science education internationally. This study aimed to explore the role of employability capital in supporting nutrition science graduate outcomes across Australia and Ireland. More specifically, it sought to answer the following research questions: Does the GCM explain the perceived employability of nutrition graduates, and how does the GCM explain the perceived employability of nutrition graduates?

## Methods

Our study took an interpretive approach focused on beliefs and experiences, whereby meaning was socially constructed through both the experiences of participants and researchers acknowledging their multiple experiences^(7)^. In addition, the GCM was used as a theoretical concept to assist in interpreting results. Our research team consisted of an experienced qualitative researcher and dietitian (CP), an experienced nutrition science researcher (LR) and two junior nutrition researchers with training in qualitative research (CB and SOD). The novice researchers kept reflexive journals throughout the interview process.

### Participants

The study was conducted with graduates from two countries – Australia and Ireland, selected due to similar education systems and nutrition science training. The graduates in Australia had minimum bachelor-level nutrition science degrees at a single, large, research-intensive university in Melbourne. They were interviewed between July and September 2021. The Irish participants had minimum honours-level nutrition science degrees at eight Higher Education Institutions in Ireland and were interviewed between January and February 2022. At the time of the interviews, participants were between 0 and 7 years postgraduation. All degrees in both countries were accredited or in the process of seeking accreditation with the Association for Nutrition (AfN), an independent regulator of nutritionists in the UK who provide voluntary individual and nutrition degree accreditation using a system based on core competency areas and set standards for training and education^(8,9)^. The structure of the degrees differs slightly, as Irish nutrition science degrees include an embedded honours year, while the Australian degree does not. Participants were eligible if they had completed their undergraduate degree between 2015 and 2021 and had not studied, nor were enrolled to study dietetics. Graduates doing postgraduate study in any area besides dietetics were also eligible to participate. Although both Australian and Irish universities offered an elective work-integrated learning experience, students did not have to have completed this to be included in our study.

In both cohorts, the concept of information power underpinned the maximum variation sampling technique in line with our interpretivist approach. Despite the strong theoretical foundation of the study’s topic, high quality of discussions and framework analysis strategy, given the broad aim of the study and sample specificity, a moderate sample size was deemed adequate^(10)^. Researchers aimed to recruit at least fifty participants across the two countries who had diverse employment stories. This could include, but was not limited to, employment experiences in a variety of fields, and/or the decision to complete further study.

Participants were initially recruited via a promotional post on LinkedIn, and then to support the achievement of maximum variation, eligible individuals were contacted via personal LinkedIn messages. Those interested were prompted to complete an expression of interest form. The form, which was identical across both groups, asked for the participant’s full name, email address, the university at which they completed their undergraduate degree, year of study completion and whether they had completed or were currently enrolled in dietetics. Sixty-eight (*n* 16 Australian; *n* 52 Irish) graduates responded to the initial posts, fifty-eight (*n* 15 Australian; *n* 43 Irish) met the eligibility criteria and forty-one (*n* 13 Australian; *n* 28 Irish) participated in an interview (four withdrew prior to the interview citing lack of time, and thirteen did not respond to the follow-up email). An additional thirty-five (*n* 15 Australian; *n* 20 Irish) participants were purposefully messaged by the first authors to increase the sample size and twenty-three (*n* 9 Australian; *n* 14 Irish) responded and consented to participate. At this point in data collection, the researchers believed the sample provided adequate depth and breadth to answer the research questions. A range of graduate employment and employability experiences were captured, so researchers decided to stop any further recruitment.

### Interview protocol

Narrative interviews were chosen for data collection as they provided an opportunity for the participant to share in-depth stories of their experiences in a chronological order, from their time as a student to entering the workforce, to the present, as a professional^(11,12)^. All interviews were conducted over an online meeting platform by either CB (Australia) or SOD (Ireland). A series of semi-structured interview questions were designed to associate each narrative prompt, to ensure that all interviewers covered the same points and was similar across countries (Table 1). Questions were centred around the three time points of pre-graduation (including time on placement, if applicable), immediately after graduation, and now, as a professional. Each interview lasted between 25 and 100 min (M = 45·80 min, sd = 14·60 min) in duration and was transcribed verbatim. Participants were given vouchers to compensate for their time.

**Table 1:** Interview guide for exploring nutrition science graduates’ experiences of employment and employability

Time point	Narrative prompt	Example questions for participant
Before graduation	Prompt the participant to recount their story of entering nutrition study, including their highlights, lowlights, mentors and any extra or co-curricular endeavours.	Why did you decide to study nutrition? What did you intend to do with your degree, in terms of employment? What were the highlights and lowlights of your time as a student? Did you do placement/research unit or electives in third year? Expand on your experience. Did you do anything outside your studies (part time work, extra/co-curricular activities?) Is there anything you can think of that enhanced your employability? Did you have any mentors as a student that guided your entry into the workforce?
Immediately after graduation	Prompt the participant to recount their story as a Bachelor of Nutrition Science graduate, including considerations about further study, job seeking and perceptions of preparedness.	Talk me through what happened once you finished your degree. Why do you think you got the graduate role you have? Which aspects, if any, of your nutrition degree do you use in your current role? Did you consider further study pathways? What was the job seeking experience like for you? How prepared did you feel to enter the workforce? Walk me through a day in your life in the current job you are in.
Now, as a professional	Prompt the participant to recount their story as a professional, including their current working arrangements, professional identity and future plans.	Now that you are working as a nutrition science graduate, what does it mean to you to be a nutrition professional? Tell me about a specific time when you felt like a nutrition professional. Do you feel a sense of professional identity, that is, a sense of professional belonging in the nutrition field? What are your future plans? What advice would you give to students about to enter the first year of their nutrition degree? What advice would you give to a new grad about to enter the job market?

### Data analysis

Data were narratively analysed in two stages, where the analysis focused on the description of participants’ stories of employment and employability. First, two authors independently and inductively open-coded the transcripts and then came together to compare and refine to a single set of codes and grouped them into categories. This initial stage allowed an in-depth interpretation of the dataset to facilitate the data analysis against the GCM. Second, the data were then deductively coded against the five graduate capitals (human, social, identity, cultural and psychological) in the GCM, and the patterns and frequency of codes against each capital in the model noted to identify how elements of capital explain employability. The original analysis was used to assist description of the coding against the CGM. During the analysis process, the two authors (CB and SOD) came together regularly to compare coding and data analysis. Selected quotes from Australian and Irish cohorts were then chosen to illustrate the capitals.

## Results

Sixty-four graduates participated in this study across both countries. The majority of participants were female (93·7 %), currently employed and not currently studying at the time of being interviewed (Table 2). Almost half of the graduates completed their degrees between late 2019 and 2021 (*n* 31 or 48 %), during the COVID-19 pandemic.

**Table 2:** Characteristics of nutrition science graduates (*n* 64) interviewed in this study, Australia and Ireland

Attribute	Australian participants		Irish participants		Total number of participants	
	*n*	%	*n*	%	*n*	%
Gender						
Female	21	32·8	39	60·9	60	93·7
Male	1	1·6	3	4·7	4	6·3
Year of graduation						
2015	5	7·8	1	1·6	6	9·4
2016	3	4·7	6	9·4	9	14·1
2017	2	3·1	5	7·8	7	10·9
2018	4	6·3	7	10·9	11	17·2
2019	4	6·3	7	10·9	11	17·2
2020	3	4·7	3	4·7	6	9·4
2021	1	1·6	13	20·3	14	21·9
Residence (province)						
Victoria, Australia	18	28	–		18	28
New South Wales, Australia	1	1·6	–		1	1·6
Connacht, Ireland	–		8	12·5	8	12·5
Leinster, Ireland	–		20	31·3	20	31
Munster, Ireland	–		9	14·1	9	14·1
Ulster, Ireland	–		4	6·3	4	6·3
Overseas	3	5	1	1	4	6·3
Placement experience						
Completed placement	13	20·3	37·	57·8	50	78·1
Did not complete placement	9	14·1	5·	7·8	14	21·9
Education – Australia						
BNutSc	14	21·9	–		14	21·9
BNutSc (Hons)	3	4·7	–		3	4·7
BNutSc + undergraduate degree	3	4·7	–		3	4·7
BNutSc + postgraduate degree	2	3·1	–		2	3·1
Education – Ireland						
BSc (Hons) Human Nutrition	–		16	25	16	25
BSc (Hons) Public Health Nutrition	–		6	9·4	6	9·4
BSc (Hons) Nutritional Sciences	–		4	6·3	4	6·3
BSc (Hons) Nutrition and Health Science	–		7	10·9	7	10·9
BSc (Hons) Nutraceuticals in Health and Nutrition	–		1	1·6	1	1·6
BSc (Hons) + MSc in Nutrition	–		6	9·4	6	9·4
BSc (Hons) + PhD in Nutrition	–		2	3·1	2	3·1
Graduates currently studying						
Not currently studying	15	23·4	30·	46·9	45	70·3
Master of Public Health	5	7·8	–		5	7·8
Master of Nutrition	–		6	9·4	6	9·4
PhD in Nutrition	1	1·6	5	7·8	6	9·4
Other^*^	1	1·6	1	1·6	2	3·1
Current employment status						
Employed – full-time	20	31·2	27	42·2	47	73·4
Employed – part-time	0	0	7	10·9	7	10·9
Unemployed/seeking work	2	3·1	8	12·5	10	15·6
Main area of employment since graduating						
Food industry	6	9·4	12	18·7	18	28·1
Research	5	7·8	8	12·5	13	20·3
Health promotion	3	4·7	1	1·6	4	6·3
Administration	2	3·1	2	3·1	4	6·3
Academia	0	0	2	3·1	2	3·1
Self-employed/freelance nutrition	1	1·6	2	3·1	3	4·7
Performance nutritionist	0	0	2	3·1	2	3·1
Other^†^	2	3·1	8	12·5	10	15·6
Unemployed/seeking work	2	3·1	6	9·4	8	12·5

* Currently studying: Master of Education (1), Undergraduate (1).

† Other area of employment: regulatory affairs – supplements (1), regional project manager (1), science communications (1), physician associate (1), toxicologist (1), Scientific writer in pharmaceuticals (1), accountancy (1), clinical assistant (1), data management (1), sales consultant (1).

### Does the graduate capital model explain the perceived employability of nutrition graduates?

All elements of the GCM were identified in the data. Human, social and identity capital were identified as most dominant within the data as influential on employment outcomes (Table 3) and therefore are elaborated further below. For the three dominant capital present in the data – Human Capital, Social Capital and Identity Capital – either a positive or negative association with the development of each capital was identified amongst all participants.

**Table 3:** Mentions of graduate capital by frequency

Capital	Explanation of capital	Australia		Ireland		Total		Example quote
		*n*	%	*n*	%	*n*	%	
+ Human capital	Research skills, nutrition knowledge, food regulation and composition knowledge useful in the workforce	16	72	35	83	51	80	‘Being able to summarise…data and to communicate is something I think most jobs require. I think I was well practiced in the nutrition degree’ (A9F2015) ‘but I think it, I always know that I have this knowledge. I have this evidence-based knowledge and it’s the best evidence that there is out there’ (I001F2019)
− Human capital	Knowledge gained in the degree had limited use in the workforce	6	27	7	17	13	20	‘I don’t really think my degree helped. It’s really irrelevant to what I’m doing, which kind of pisses me off’ (A7F2019) ‘My Mum asked me… ‘how much of your knowledge of bachelors are you using right now?’ And I replied, ‘2 % tops!’ It’s so little’ (A1F2020) ‘I feel like I haven’t been able to keep up with the knowledge for the past three years. And so I feel like I don’t have that information to provide any more’ (I018F2019)
+ Social capital	Mentors in the field supporting mentees with advice, links to job opportunities, online network building and work experience	19	86	30	71	49	77	‘You may have the perfect grades. But if you have the right connections, you’re in a better place like, you know, the more people you know in the industry, the more offers and opportunities there are for you up there’ (A13F2019) ‘but I did a lot of work with the Nutrition Society. So I think yeah, so I think I purposely got involved in the nutrition side and things, so I definitely had an amazing sense of community and belonging in the nutrition field and I got to network a lot’ (I022F2017)
− Social capital	Lack of mentorship, connections to the nutrition profession	3	14	13	31	16	25	‘I think…a mentor would have helped…to bounce things off and just to tell them how it’s how I was feeling. I think knowing someone in the industry would have helped outside of uni… maybe, like, alumni who would have gone through the same thing?’ (A3F2015)
+ Identity capital	Presence of a professional narrative, an ‘employable self’, feels like a nutrition professional	9	41	34	81	43	67	‘I think…there is this level of professionalism that I carry around in the work that I do. This role recognises my qualifications, to be able to say because you have a nutrition science degree, you understand the basics of nutrition’ (A6F2018)
− Identity capital	Does not feel a sense of professional identity Contention with non-degree qualified nutritionists Hasn’t worked as a nutritionist, so not a nutrition professional	13	59	8	19	21	32	‘I actually don’t [feel professional identity], I wish I did. And I think if I were to do dietetics, I would have…I always wish that there was almost something else we could call ourselves like, accredited. I don’t know, something…other people would know that we actually have done a degree in this as opposed to just a certificate’ (A21F2018) ‘honestly no. I feel major impostor syndrome’ (I023F2018)
+ Cultural capital	Cultural exposure to the nutrition field Interpersonal and behavioural expectations	5	23	12	29	17	27	‘I do feel like I was a professional at [placement company]. And I did belong there. You feel like you’re doing your job’ (A7F2019)
+ Psychological capital	Sense of resilience, adaptability, openness to experience and risk tolerance Flexible contingency planning	3	14	9	21	12	19	‘I think [what got me the job] is a combination of grit and resilience…I think probably relying on strengths as well is probably another thing and focusing on those things rather than putting yourself down with what you think you might not be so good at’ (A3F2015)

+ denotes graduates who mentioned having the capital and − denotes graduates who mentioned not having the capital.

#### Human capital

The presence of human capital, possession of hard skills and specialist knowledge, was high amongst the joint cohort with four out of five graduates reporting having attained human capital from their nutrition science programmes. Graduates across both countries elaborated on their sound nutrition knowledge, reporting that they left their degrees feeling confident in having attained the knowledge and technical skills they needed to work (Table 3, I001F2019). Research skills were reported most often as being well taught throughout the respective degree programmes and graduates reported feeling prepared to carry out critical research and differentiate between good and poor-quality research.

*‘the research like traits that I learned…were a massive standpoint to me. I definitely think it helped like shaping like discipline and like tuning my eye into data’ (I036F2019)*

A small number reported that they did not gain sufficient knowledge for application to the workforce or that work in a non-nutrition-specific sector resulted in their knowledge no longer being up to date with the latest evidence. One participant described their experience of being in a job that was not directly utilising their nutrition science knowledge and over time they perceived this diminished their capacity to recall and utilise that specialist nutrition knowledge with confidence (Table 3, I018F2019). Another graduate reported believing the knowledge and skills developed during their nutrition science degree was not useful in the job seeking process and was angered by this.

*‘I don’t really think my degree helped. It’s really irrelevant to what I’m doing, which kind of pisses me off’ - (A7F2019)*

#### Social capital

Developing networks and building relationships within the nutrition field was reported by 77 % of total participants as crucial for enhancing employment prospects and maintaining an awareness of opportunities for personal and professional development. Presence of social capital was similar across countries. Participation in conferences, nutrition societies and other organisations, such as charities and community groups, was perceived by participants as providing great opportunities to network and form lasting relationships with contacts in the field (Table 3, I022F2017). They described a strong network and the ability to form connections with other nutrition professionals across the various employment sectors as essential for boosting chances of securing employment.

*‘You may have the perfect grades. But if you have the right connections, you’re in a better place like, you know, the more people you know in the industry, the more offers and opportunities there are for you up there’ (A13F2019)*

Approximately one-quarter of participants described a lack of social capital, reporting few connections built up within the nutrition field and absence of a support network in nutrition. Many of these participants perceived that having a mentor in nutrition would have greatly helped in this regard to discuss career opportunities, provide connections to relevant professionals in the workforce and support them in their career progression. However, these same participants also stated that they did not seek mentorship relationships or pursue opportunities to foster future mentor–mentee connections.

*‘I think…a mentor would have helped…to bounce things off and just to tell them how it’s how I was feeling. I think knowing someone in the industry would have helped outside of uni… maybe, like, alumni who would have gone through the same thing?’ (A3F2015)*

#### Identity capital

The formation of a professional identity, presence of a professional narrative and connection to the nutrition profession field was strongest in the Irish graduates with 81 % of graduates describing identity formation, compared with the Melbourne cohort with 41 %. Over three-quarters of Irish graduates expressed a connection to the nutrition field, feeling like a nutrition professional, and reported a sense of belonging within the nutrition field. Whilst thirteen out of twenty-two Australian participants cited a lack of connection or identity.

*‘I loved like working in this space ’cause you did feel like you know we’re doing something kind of good here …. So yeah, I think I ….felt definitely a sense of belonging’ (I033F2017)*

The perceived impact of nutrition on the ability to improve community and individual health and influence policy informed professional identity formation among graduates. Participants cited identity in helping people, working against the torrent of misinformation and feeling part of a trusted profession. Graduates who could identify with the wider impact of their work cited a stronger sense of professional identity.

*‘…. I think that that’s what distinguishes us from people that just have an interest or people that don’t necessarily have the qualifications we do’ (A22F2017)*

Work experience was mentioned frequently with respect to building professional identity among graduates in their time at university. Participating in a placement allowed students to be recognised as professionals and apply their theoretical nutrition knowledge to real-life projects.

*‘In my placement […] I think because you have that kind of strength and unity of a team, […] it felt really, really nice to be a part of this little team who […] knew what I was saying was correct because they had agreed with me’ (I007F2018)*

Barriers to professional identity formation throughout nutrition science education were also identified. Throughout the interview process, the concept of imposter syndrome arose many times across both cohorts (Table 3, I023F2018). Participants reported feeling unsure of their job prospects, how to leverage their capabilities to secure employment and being second class to other healthcare professionals due to a lack of clinical patient experience.

*‘I actually don’t [feel professional identity], I wish I did. And I think if I were to do dietetics, I would have…’ (A21F2018)*

The lack of regulation or title protection for suitably qualified nutrition professionals arose in 50 % of interviews. Participants explained that this lack of title protection resulted in different interpretations of the term ‘Nutritionist’ or ‘Nutrition Professional’ worldwide leading to confusion as to what a ‘nutritionist’ does, the basic qualifications they should have attained, and their scope of practice. In the absence of a clear definition, clarity surrounding career options and pathways was reported to have become diminished.

*‘I always wish that there was almost something else we could call ourselves like, accredited. I don’t know, something…other people would know that we actually have done a degree in this as opposed to just a certificate’ (A21F2018)*

A further consequence of the lack of regulation is the presence of non-degree qualified individuals using the ‘Nutritionist’ title for work. These unsuitably qualified individuals do not abide by a professional code of conduct and predominantly practise outside of an evidence base. Contention with these individuals utilising the title for their own work left many of the participants feeling like the profession is no longer a respected job, anyone can become a nutritionist. In addition to comparison with dietetics, graduates explained contending with comparisons to unqualified individuals, which has led to ‘a bit of a blurred identity’ and a challenge for graduates to distinguish themselves from others.

*‘We’re in this really weird space where we’re not a dietitian, but we’re also trying to say that we’re different from people that don’t have the same level of qualification as us’ (A22F2017)*

## Discussion

This study aimed to understand if the GCM explained the employability of nutrition graduates. The overall strong positive experience described by the graduates in developing Human, Social and Identity capital is encouraging and demonstrates that formal nutrition science education programmes are providing students with the necessary opportunities for developing employability skills and attributes. The data show that more emphasis may need to be placed on cultural and psychological capital in nutrition science education to support employability outcomes. These findings have implications for nutrition science educators internationally.

Strong human capital across both Irish and Australian graduates was unsurprising, as nutrition science curricula strive to embed the necessary specialist knowledge and skills development across programmes to ensure graduates develop required competencies to practice professionally^(8,13)^. Only one of the degree programmes mentioned by the graduates was not an AfN accredited programme at the time of the study, meaning most programmes followed a competency-based education approach whereby curricula are designed to meet AfN standards and competency requirements^(14)^. Both countries reported sound knowledge and the ability to use these skills postgraduation, which is a testament to the nutrition science education programmes. The small number who reported a negative association with human capital were predominantly working in roles where they were not using those hard skills on a regular basis and thus perceived their abilities were waning as more time passed. While these data are positive, the literature would suggest that modern employers and stakeholders are moving away from solely looking for hard skill acquisition when considering prospective employees, placing importance on other aspects including personal attributes and values^(14)^. Nutrition education programmes must not lose sight of its strong science foundations but ensure attention is paid to other skills development.

Social capital was equally strong amongst graduates in both countries. In today’s modern society, the concept of networking is considered of high importance for career progression^(15)^. Nutrition graduates in Ireland and Australia are familiar with this concept and are exposed to the different ways for building networks from an early stage in their teaching and learning. Formation of relationships through networking with colleagues and peers has also been shown to be advantageous for sharing of knowledge and resources^(16,17)^. The lack of social capital described by a quarter of participants, predominantly Irish graduates, is likely a consequence of the pandemic, during which many Irish graduates completed their programmes. The pandemic resulted in the cancellation of in-person networking events, such as conferences, and the enforcement of virtual means of contact for communication and building relationships. This period of social isolation left many individuals feeling alone and disconnected^(18)^. This prolonged isolation and the challenge of fostering meaningful relationships via virtual means of communication could potentially have impacted these young graduates’ capabilities to network and establish themselves within the local and national nutrition communities resulting in a lack of social capital. Our findings suggest that opportunities for networking and connections to the nutrition science community should be built into standard curriculum and not be electives or volunteer opportunities.

The Irish cohort strongly held a sense of identity capital compared with Australian graduates. The Nutrition Society, formed in 1941, which is a long-established organisation in the UK was created to form a community of nutrition professionals to support one-another and challenge existing ideas to bring about progress within the nutrition field. In 1988, an Irish Section committee was established to develop a strong sense of community amongst those in the nutrition field and strengthen the ties between the Irish and UK networks^(19)^. Additionally, the growing presence and recognition of the AfN across Ireland and the UK have also strengthened the nutrition community in Ireland – by providing an accreditation system for qualified nutritionists, and they have provided accountability, credibility and a sense of security in the absence of title regulation^(20)^. As of 2022, the AfN has over 4000 members registered to the UK Voluntary Register of Nutritionists (UKVRN) and ninety-nine accredited programmes^(21)^. Of the nine Irish undergraduate (honours) bachelor’s degrees the graduates completed, eight were accredited with the AfN at the time of the study. These graduates were exposed during their degree to the purpose of the AfN and the benefits of accreditation^(13)^, further strengthening their sense of connection with the greater network of nutrition professionals across Ireland and the UK.

Completion of placement during the degree (90 % of Irish and 59 % of Australian graduates) differed between both countries and is potentially a contributing factor in the presence of positive professional identity among the Irish graduate cohort. Placement experience, or work-integrated learning, is implemented on health profession education curricula to provide students with real-life experience of work settings and an opportunity to further develop a sense of professionalism through translation of their knowledge and skills to workplace tasks^(22,23)^. This experience widens students’ understanding of career opportunities and perceptions of life as a nutrition professional. A study on dietetics graduates’ experiences by Morgan *et al.* (2020) highlighted the importance of well-structured work-integrated learning experiences to prepare students for the workforce^(24)^. Practical experience via a placement improves students’ experiences in transitioning to the workforce and enhances their employment prospects and should be considered a mandatory part of all nutrition science education.

Identity guides behaviour, and in a professional context, fosters confidence (both in oneself, and clients in the professional)^(25)^. Professional identities are broadly defined as how an individual perceives themselves as a professional based on factors such as their values, beliefs and experiences in relation to their profession^(26)^. A recent qualitative exploration of the future of nutrition and dietetics in Australia and New Zealand calls for the development of a cohesive profession with a strong identity^(27)^. This work emphasises the need to embrace nutritionists and nutrition scientists so that the entire profession is underpinned by clear credentialing, accreditation and supervision. Robust, regulatory frameworks can also enable professional movement across international borders and work towards a global understanding of the roles of nutrition scientists, nutritionists and dietitians. It is evident that accreditation of courses, and registration of graduates, may bolster the development of identity capital.

The sample was large and diverse, supporting the credibility of the findings. However, despite the sample including diverse experiences of graduates, the findings may not be transferable to all Australian and Irish nutrition science graduates’ experiences. Several graduates interviewed in this study are likely to have had their job search impacted by COVID-19 (graduates from the end of 2019 onwards), which may explain the difficulty experienced in securing work. Despite this limitation, the participants who graduated pre-pandemic echoed similar struggles with obtaining work as a graduate, indicating that these challenges have likely been exacerbated, rather than caused by, COVID-19.

We observed limitations in the GCM during application of the model to our findings. First, the model is individualistic and does not take into account the broader social and economic context in which graduates operate. For example, economic downturns or shifts in industries can significantly affect graduates’ job prospects, regardless of their possession of capitals. Second, the GCM oversimplifies the complex interplay between education, individual abilities and other factors such as social class, race and gender, which influence an individual’s career outcomes. Accessibility of graduate capitals is not uniform, as it is influenced by power dynamics and social status within a specific field or social context. The constraints of the model’s implementation underscore the significance of engaging in collaborative efforts with universities and employers to tackle the disparities in the manner in which students navigate the shift from education to employment. However, it has been useful in identifying targets for education development to support employment and employability of nutrition science graduates internationally.

In conclusion, the findings from this multi-institutional study indicate that formal nutrition science education programmes build human capital by providing students with opportunities to develop essential skills and attributes. However, cultural and psychological capital could be expanded to support employability outcomes. Additionally, work-integrated learning experiences, such as placements, should be considered mandatory to enhance students’ transition to the workforce and development of both social and identity capital. Overall, this study highlights a need for further research on quantifiable graduate employment outcomes, cultural and psychological capital development in nutrition science education, and consideration to employability through competency standards and curriculum.
